# The Heat Sensing Trpv1 Receptor Is Not a Viable Anticonvulsant Drug Target in the *Scn1a* ^*+/−*^ Mouse Model of Dravet Syndrome

**DOI:** 10.3389/fphar.2021.675128

**Published:** 2021-05-17

**Authors:** Vaishali Satpute Janve, Lyndsey L. Anderson, Dilara Bahceci, Nicole A. Hawkins, Jennifer A. Kearney, Jonathon C. Arnold

**Affiliations:** ^1^ Lambert Initiative for Cannabinoid Therapeutics, Brain and Mind Centre, Sydney, NSW, Australia; ^2^ Discipline of Pharmacology, Sydney Pharmacy School, Faculty of Medicine and Health, The University of Sydney, Sydney, NSW, Australia; ^3^ Department of Pharmacology, Feinberg School of Medicine, Northwestern University, Chicago, IL, United States

**Keywords:** SB-705498, epilepsy, seizures, epileptic encephalopathies, SCN1A gene, Cannabidiol (CBD), dravet syndrome (SMEI), the transient receptor potential vanilloid 1

## Abstract

Cannabidiol has been approved for the treatment of drug-resistant childhood epilepsies including Dravet syndrome (DS). Although the mechanism of anticonvulsant action of cannabidiol is unknown, emerging data suggests involvement of the transient receptor potential cation channel subfamily V member 1 (Trpv1). Pharmacological and genetic studies in conventional seizure models suggest Trpv1 is a novel anticonvulsant target. However, whether targeting Trpv1 is anticonvulsant in animal models of drug-resistant epilepsies is not known. Thus, we examined whether Trpv1 affects the epilepsy phenotype of the F1.*Scn1a* ^*+/−*^ mouse model of DS. We found that cortical *Trpv1* mRNA expression was increased in seizure susceptible F1.*Scn1a* ^*+/−*^ mice with a hybrid genetic background compared to seizure resistant 129.*Scn1a* ^*+/−*^ mice isogenic on 129S6/SvEvTac background, suggesting *Trpv1* could be a genetic modifier. Previous studies show functional loss of Trpv1 is anticonvulsant. However, Trpv1 selective antagonist SB-705498 did not affect hyperthermia-induced seizure threshold, frequency of spontaneous seizures or survival of F1.*Scn1a* ^*+/−*^ mice. Surprisingly, *Trpv1* deletion had both pro- and anti-seizure effects. *Trpv1* deletion did not affect hyperthermia-induced seizure temperature thresholds of F1.*Scn1a* ^*+/−*^; *Trpv1* ^*+/−*^ at P14-16 but was proconvulsant at P18 as it reduced seizure temperature thresholds. Conversely, *Trpv1* deletion did not alter the frequency of spontaneous seizures but reduced their severity. These results suggest that *Trpv1* is a modest genetic modifier of spontaneous seizure severity in the F1.*Scn1a* ^*+/−*^ model of DS. However, the opposing pro- and anti-seizure effects of *Trpv1* deletion and the lack of effects of Trpv1 inhibition suggest that Trpv1 is unlikely a viable anticonvulsant drug target in DS.

## Introduction

Dravet syndrome (DS) is a catastrophic early onset epileptic encephalopathy that typically begins with febrile seizures and progresses into numerous spontaneous afebrile seizures that are poorly managed by currently available anticonvulsant drugs (Scheffer et al., 2017). The majority of DS patients have loss-of-function *de novo* mutations in the *SCN1A* gene that encodes the alpha subunit of type I voltage-gated sodium channel Na_v_1.1 (Marini et al., 2011; Brunklaus and Zuberi, 2014). However, *SCN1A* mutations have varying penetrance and phenotypic severity (Gambardella and Marini, 2009), thought to result from variants in genetic background modifying the impact of the *SCN1A* mutation, known as genetic modifiers. From a drug discovery perspective, the identification of genetic modifiers may provide novel anticonvulsant drug targets.

Mice with heterozygous deletion of *Scn1a* (*Scn1a* ^*+/−*^) recapitulate key clinical features of DS including increased sensitivity to febrile seizures, early onset spontaneous seizures, reduced survival, (Miller et al., 2014), and cognitive and behavioral deficits (Bahceci et al., 2020). *Scn1a* ^*+/−*^ mice on the 129S6/SvEvTac background (129.*Scn1a* ^*+/−*^) are seizure resistant and do not display an overt seizure phenotype, while those on the [129S6/SvEvTac × C57BL/6J]F1 background (F1.*Scn1a* ^*+/−*^) are seizure-susceptible and display a severe seizure phenotype with premature mortality (Miller et al., 2014). This strain-dependent phenotype severity suggests that the introduction of the C57BL/6J genetic background modifies the impact of the heterozygous deletion of *Scn1a,* and enhances the expressivity of the severe seizure phenotype*.* Potential modifier genes can then be inferred by comparing the coding sequence and expression of a candidate gene between seizure susceptible and seizure resistant mouse strains.

Cannabidiol (CBD), the major non-psychoactive component of cannabis plant, is a first in class FDA-approved drug for treating DS (Devinsky et al., 2017a; 2017b; Cross et al., 2017). While the mechanism underlying the anticonvulsant action of CBD is unknown and likely multimodal, emerging evidence suggests that Trpv1 receptors may contribute to the anticonvulsant effects of CBD. Accordingly, the anticonvulsant effects of CBD were reversed by a Trpv1 antagonist in the PTZ model (Vilela et al., 2017) and reduced in *Trpv1* ^*−/−*^ mice compared to wildtype mice in the MES model (Gray et al., 2020). These results are consistent with cellular studies which show that CBD activates and subsequently desensitizes Trpv1 receptors (De Petrocellis et al., 2011; Iannotti et al., 2014; Anand et al., 2020).

It is biologically plausible that the Trpv1 receptor is a novel anticonvulsant drug target, as these cation channels depolarize neurons in response to various stimuli including heat, low pH, lipids including the endocannabinoid anandamide, and vanilloids such as capsaicin (Caterina and Julius, 2001). Further, Trpv1 receptors can modulate both glutamatergic (Marinelli et al., 2003) and GABAergic transmission in the brain (Gibson et al., 2008), and directly interact with GABA_B_ receptors (Hanack et al., 2015). Pharmacological and genetic validation studies in conventional rodent seizure models reinforce the view that Trpv1 is an anticonvulsant target. Trpv1 receptor inhibition is anticonvulsant in experimentally induced seizure models such as the PTZ (Jia et al., 2015), 6-Hz (Socała et al., 2015), 4-AP (Gonzalez-Reyes et al., 2013) and MES models (Chen et al., 2013). In contrast, Trpv1 activation induces tonic-clonic seizures (Jia et al., 2015) and promotes febrile seizures (Kong et al., 2019) in adult mice and rats. Further, Trpv1 receptor expression is increased in the brain of temporal lobe epilepsy patients (Sun et al., 2013) and in animal models of temporal lobe epilepsy (Bhaskaran and Smith, 2010).

Collectively, these studies suggest that Trpv1 receptor inhibition is a viable strategy for reducing seizures in conventional epilepsy models. However, it is unknown whether Trpv1 is an anticonvulsant drug target in animal models of drug-resistant epilepsies. Thus, we compared *Trpv1* mRNA expression between seizure susceptible and seizure resistant genetic background strains of *Scn1a* ^*+/−*^ mice. We then examined whether pharmacological blockade of Trpv1 receptors or heterozygous deletion of *Trpv1* is anticonvulsant in the F1.*Scn1a* ^*+/−*^ mouse model of DS.

## Materials and Methods

### Mice

All animal care and experimental procedures were approved by the University of Sydney Animal Ethics Committee and were in agreement with the Australian Code of Practice for the Care and Use of Animals for Scientific Purposes. *Scn1a* ^*+/−*^ mice were purchased from the Jackson Laboratory (*Scn1a* ^tm1Kea^, stock 37107-JAX; Bar Harbor, MA, United States) and maintained by continuous backcrossing to 129S6/SvEvTac (129.*Scn1a* ^*+/−*^). Experimental mice were generated by crossing 129.*Scn1a* ^*+/−*^ mice with wildtype (WT) C57BL/6J mice (Australian Resources Center, stock 000664), resulting in *Scn1a* ^*+/−*^ and WT mice on a [129S6/SvEvTac × C57BL/6J]F1 background, abbreviated as F1.*Scn1a* ^*+/−*^ and F1.WT, respectively. The *Scn1a* genotype was determined as previously described (Miller et al., 2014). For *Trpv1* genetic knockout studies *Trpv1* ^+/−^ mice were kindly provided by Prof Bernard W Balleine (Caterina et al., 2000; Shan et al., 2015). The 129.*Scn1a* ^*+/−*^ mice were crossed with *Trpv1* ^+/−^ mice on a congenic C57BL/6J background generating mice on an [129S6/SvEvTac × C57BL/6J]F1 background abbreviated as F1.*Scn1a* ^+/−^; *Trpv1* ^+/−^. F1.*Scn1a* ^*+/−*^ and F1.*Scn1a* ^+/−^; *Trpv1* ^+/−^ mice were used*.* Mice were group housed in a specific pathogen-free mouse facility with food and water available *ad libitum* and housed on 12-h light/dark cycle.

### Trpv1 mRNA Quantification Using Droplet Digital PCR (ddPCR)

To determine *Trpv1* mRNA expression we performed RT-ddPCR using Taqman assays using a QX200 Droplet Digital PCR System (Bio-Rad, Hercules, CA, United States). First-strand cDNA was synthesized from 2 μg of RNA using oligo (dT) primer and Superscript IV reverse transcriptase (RT) according to the manufacturer’s instructions (Life Technologies). Quantitative ddPCR was performed using ddPCR Supermix for Probes (No dUTP) (Bio-Rad) and TaqMan Gene Expression Assays (Life Technologies) for mouse *Trpv1* (FAM-Assay Mm01246302) and *Tbp* (VIC-MGB-Mm00446971_m1) as a normalization standard. Reactions were partitioned into droplets using a QX200 droplet generator (Bio-Rad). PCR conditions were 95°C for 10 min, then 44 cycles of 95°C for 30 s and 60°C for 1 min (ramp rate of 2°C/s) and a final inactivation step of 98°C for 5 min. Following amplification, droplets were analyzed with a QX200 droplet reader with QuantaSoft v1.6.6 software (Bio-Rad). Relative transcript levels were expressed as a ratio of Trpv1 concentration to Tbp concentration.

### Pharmacokinetic Analysis

A pharmacokinetic study of SB-705498 (BLDpharm, Catalog # BD 300171, MW = 429.23) was performed to determine the target experimental time point for subsequent hyperthermia-induced seizures. Male and female mice postnatal day 21–28 (P21-28) were administered a single intraperitoneal (i.p.) injection of 1 mg/kg SB-705498 in a volume of 10 ml/kg. At selected time points (15, 30, 60, 90, 120 min; four to five mice/time point) following the injection blood and whole brains were collected. Plasma was isolated by centrifugation (9,000 *g* for 10 min). Plasma and brain samples were stored at −80°C until analyzed as described below. Plasma and brain SB-705498 concentrations at each time point (Supplementary Figure S1).

### Hyperthermia-Induced Seizures

Hyperthermia-induced seizures were performed in male and female *Scn1a* ^*+/−*^ mice P14-16 as previously described (Hawkins et al., 2017). For pharmacological studies mice were randomly injected with vehicle or SB-705498 (10 or 20 mg/kg) as a single i.p. injection ∼15 min prior the onset of hyperthermia-induced seizure as the Tmax of SB-705498 was 15 min (Supplementary Figure S1). Hyperthermia was induced by increasing the body temperature of mice using a heat lamp connected to a temperature controller (TCAT-2DF, Instruments, Inc. Clifton, NJ, United States). Core body temperature was measured using a RET-3 rectal probe (Physitemp Instruments) and temperature was elevated 0.5°C every 2 minutes until a seizure occurred or 42.5°C was reached. Mice that reached 42.5°C and held for 3 min without generalized tonic-clonic seizure (GTCS) were considered seizure free. Onset of the first GTCS with loss of posture was noted as the seizure threshold temperature. Following the hyperthermia-induced seizure protocol, brain samples were isolated and stored at −80°C until analyzed. The experimenter was blinded to the genotype or drug condition. Hyperthermia-induced seizure threshold was analyzed using log-rank (Mantel–Cox) test, and *p*<0.05 was considered statistically significant.

### Spontaneous Seizures

A single and brief hyperthermia-induced GTCS was induced in male and female *Scn1a* ^*+/−*^ mice at P18 as previously described (Hawkins et al., 2017). Following the GTCS mice were immediately cooled to ∼37°C on a cooling pad to terminate the seizure. After recovery, mice were housed in groups of up to three in clear plexiglass chambers with mice used in the pharmacological studies randomly assigned to control or SB-705498 treatment groups. Drugs were administered orally through supplementation in chow formulated in house using R&M Standard Diet (Specialty Feeds; Glen Forrest, AUS) irradiated powder with a formulation of 500 mg SB-705498/kg chow. Mice consume chow (i.e., drug) in proportion to their body weight thus each mouse self-administers an equal dose across the treatment group (Bachmanov et al., 2002). Weight gained between P18 and P22, i.e., before the induction of hyperthermia-induced seizure and after the spontaneous seizure monitoring ensured drug administration (Supplementary Figure S2). Continuous video recordings from 12:00 on P19 through 24:00 on P21 were acquired. The number of spontaneous GTCS during this 60 h recording period were quantified offline by an observer blinded to genotype or drug treatments. Presence of a GTCS with loss of posture was scored as positive. The severity of each seizure was determined based on whether or not the seizure progressed to full hindlimb extension, which is the most severe stage of GTCS. Statistical comparisons were made using Mann–Whitney rank-sum test (spontaneous seizure frequency) or Fisher’s exact test (proportion of mice seizure-free and spontaneous seizure severity), with *p*<0.05 considered as statistically significant.

### Survival

Following the spontaneous seizure protocol, mice continued drug treatment to P30 to monitor survival. Data were analyzed using log-rank (Mantel–Cox) test with *p*<0.05 considered as statistically significant.

### Analytical Chemistry

SB-705498 concentrations in plasma and brain were assayed by LC-MS/MS based on previously described methods (Anderson et al., 2019). Plasma samples (50 µl) were spiked with internal standard (2 μg/ml diazepam, 5 µl) and then vortex-mixed with methanol and −20°C chilled acetonitrile (1:14, v/v). The organic layer was isolated by centrifugation (4,000 g for 10 min, 4°C) and evaporated to dryness with N_2(g)_. Samples were reconstituted in acetonitrile and 0.1% formic acid in water (3:10, v/v) for solid-liquid extraction with methyl tert-butyl ether using Biotage Isolute SLE + columns (Uppsala, Sweden). Samples were evaporated to dryness with N_2(g)_ and reconstituted in acetonitrile and 0.1% formic acid in water (1:1, v/v) for analysis. Brain samples were prepared as above with minor modifications. Briefly, ½ of the brain was weighed and homogenized in acetonitrile (5×, w/v). Homogenates were centrifuged (20,000 *g* for 30 min, 4°C) and 100 µl of supernatant was spiked with diazepam as internal standard (10 μg/ml, 15 µl). Extraction was achieved by vortex-mixing with 3× volume of −20°C chilled acetonitrile. The organic layer was isolated by centrifugation (20,000 *g* for 15 min, 4°C) and the supernatant was filtered using an ultracel at (20,000 *g* for 1.5 h, 4°C) evaporated to dryness with N_2(g)_. Samples were reconstituted in acetonitrile and 0.1% formic acid in water (3:10, v/v) for solid-liquid extraction as above. Samples were evaporated to dryness and reconstituted in acetonitrile and 1% formic acid in water (1:1, v/v) for analysis. Samples were assayed by LC-MS/MS using a Shimadzu Nexera ultra-high performance liquid chromatograph coupled to a Shimadzu 8,030 triple quadrupole mass spectrometer (Shimadzu Corp.; Kyoto, Japan). The mass spectrometer was operated in positive electrospray ionization mode with multiple reaction monitoring with the following mass transition pairs: *m/z* 428.95 > 258.1 (SB-705498) and 284.6 > 257.0 (diazepam). Quantification was achieved by comparing experimental samples to standards prepared with known amounts of drug.

## Results

### Seizure Susceptible *Scn1a* ^*+/−*^ Mice had Increased *Trpv1* mRNA Levels in the Cortex

**FIGURE 1:**
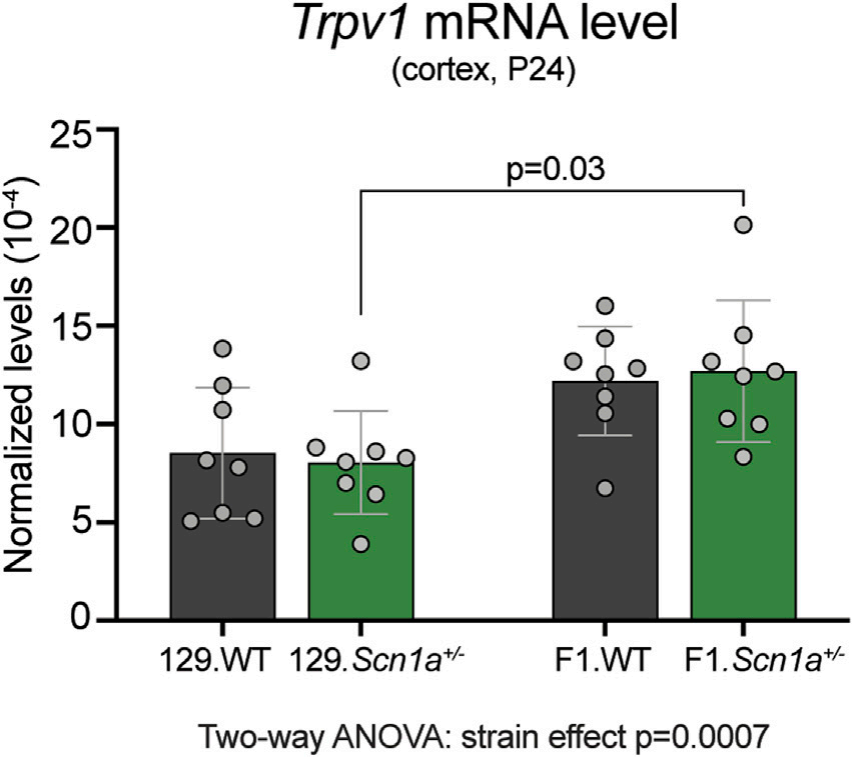
*Trpv1* mRNA expression is increased in the seizure susceptible F1.*Scn1a* ^*+/−*^ mice compared to seizure resistant 129.*Scn1a* ^*+/−*^ mice. Relative *Trpv1* transcript levels in WT (gray bars) and *Scn1a* ^*+/−*^ (green bars) mice on the seizure resistant 129 and seizure susceptible F1 (129 × B6) genetic backgrounds. Transcript levels were determined by RT-ddPCR and normalized to *Tbp. Scn1a* ^*+/−*^ mice on the F1 background had a strain- dependent increase in the cortical *Tprv1* mRNA levels compared to the wildtype and *Scn1a* ^*+/−*^ mice on the 129 background (Two-way ANOVA: strain effect *p* = 0.0007, *n* = 8 mice per group). F1.*Scn1a* ^*+/−*^ mice had significantly increased *Trpv1* mRNA levels compared to *Scn1a* ^*+/−*^ mice on the 129 background (Tukey’s multiple comparisons test *p* = 0.03).

To determine whether *Trpv1* expression may contribute to the epilepsy phenotype of *Scn1a* ^*+/−*^ mice, we compared the cortical mRNA levels from WT and *Scn1a* ^*+/−*^ mice on both 129S6/SvEvTac (129) and on [129S6/SvEvTac × C57BL/6J]F1 (129 × B6) genetic backgrounds. Mice on the seizure susceptible F1 background had significantly increased cortical *Trpv1* mRNA levels compared to the mice on the seizure resistant 129 background (strain effect *p* = 0.0007; Figure 1). In addition, the seizure susceptible F1.*Scn1a* ^*+/−*^ mice had significantly increased cortical *Trpv1* mRNA levels compared to the seizure resistant 129.*Scn1a* ^*+/−*^ mice (*p* = 0.03 with Tukey’s multiple comparisons test). This suggests that *Trpv1* could be a genetic modifier and increased *Trpv1* expression might contribute to the seizure susceptibility of F1.*Scn1a* ^*+/−*^ mice.

### Trpv1 Receptor Inhibition does not Protect Against Hyperthermia-Induced and Spontaneous Seizures in F1.*Scn1a* ^*+/−*^ Mice

**FIGURE 2:**
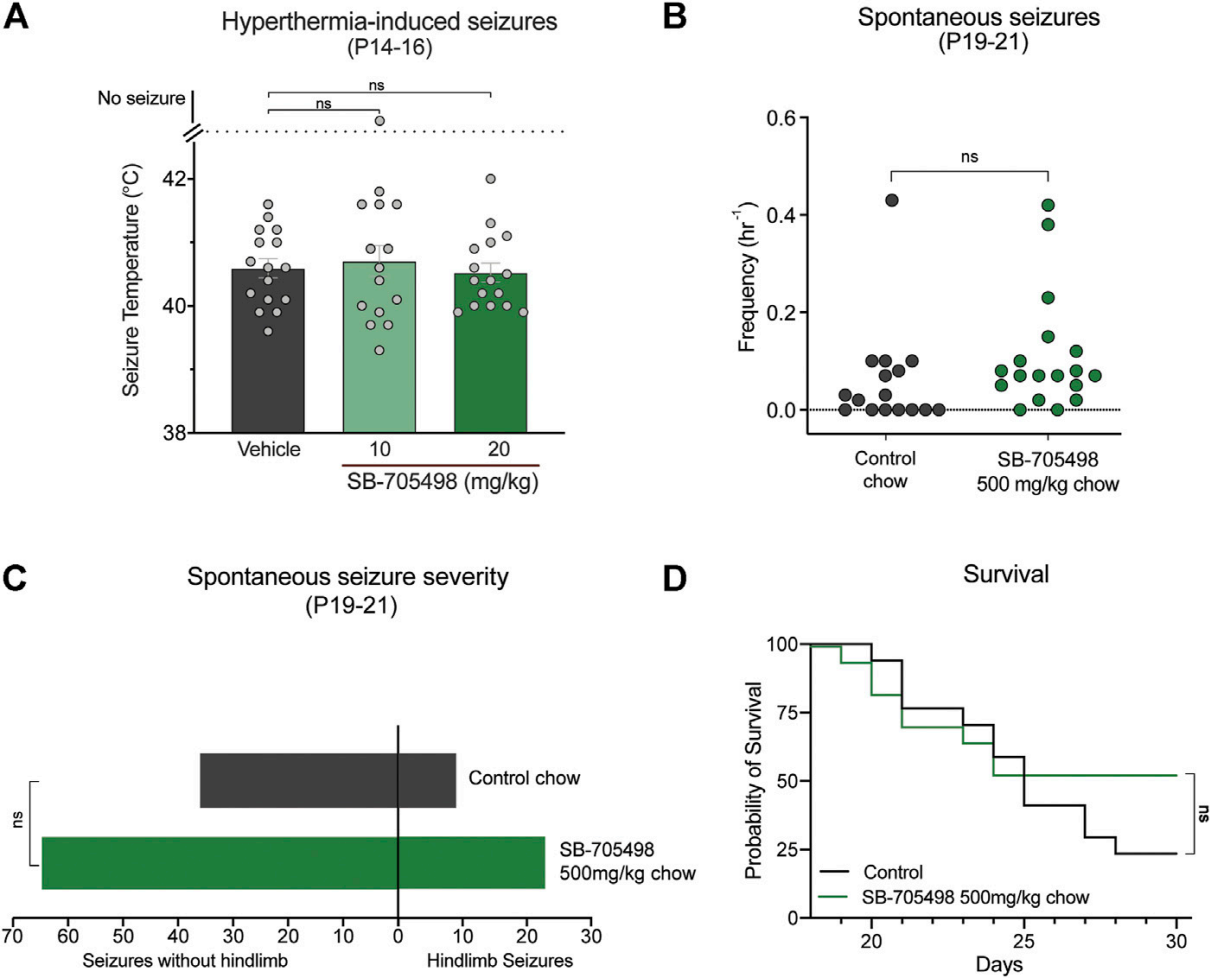
Trpv1 receptor inhibition did not have anticonvulsant effects in the F1.*Scn1a* ^*+/−*^ mice. **(A)** GTCS temperature threshold of individual P14–16 F1.*Scn1a* ^*+/−*^ mice induced by hyperthermia following acute i. p. treatment with vehicle or varying doses of SB-705498. SB-705498 had no effect on temperature thresholds. The average temperatures of seizure induction are depicted by the bars and the error bars represent SEM, with *n* = 14–16 per group [vehicle = 40.6 ± 0.15, 10 mg/kg = 40.6 ± 0.2, 20 mg/kg = 40.5 ± 0.1; *p* > 0.05, Log-rank (Mantel–Cox) test]. Effect of SB-705498 on spontaneous seizures were determined in a separate cohort of P19–21 F1.*Scn1a* ^*+/−*^ mice. **(B)** GTCS frequency of individual control-treated and SB-705498-treated F1.*Scn1a* ^*+/−*^ mice. Drug treatment administered orally through supplementation in chow was initiated following the induction of a single hyperthermia-induced GTCS on P18. Unprovoked, spontaneous GTCSs were quantified over a 60 h recording period. SB-705498 subchronic treatment did not affect spontaneous seizure frequency, with *n* = 16–18 per treatment (control chow = 0.06 ± 0.03/h, 500 mg SB-705498/kg chow = 0.11 ± 0.03/h; *p* = 0.2, unpaired *t*-test). **(C)** Number of GTCS with and without full tonic hindlimb extension is depicted. Seizure severity was not affected by subchronic SB-705498 treatment, with *n* = 16–18 per group (control chow = 9 hindlimb seizures of 45 seizures, 20%; 500 mg/kg SB-705498 supplemented chow = 23 hindlimb seizures of 88 seizures, 26%; *p* = 0.7, Chi-square test). **(D)** Survival curves comparing control-treated and SB-705498-treated mice. Treatment began at P18 and survival was monitored until P30. SB-705498 had no effect on survival of F1.*Scn1a* ^*+/−*^ mice, with *n* = 16–18 per treatment (*p* = 0.18, Log-rank Mantel-Cox).

We first sought to pharmacologically evaluate whether Trpv1 is a viable drug target in the F1.*Scn1a* ^*+/−*^ mice by assessing the effects of the selective Trpv1 antagonist SB-705498 against hyperthermia-induced seizures, spontaneous seizures, and survival of the F1.*Scn1a* ^*+/−*^ mice. Doses of SB-705498 (10 and 20 mg/kg) were chosen based on previous studies showing that 10 mg/kg was the minimum effective antinociceptive and anti-stress dose in mice (Van Den Wijngaard et al., 2009; Tékus et al., 2010). Neither 10 or 20 mg/kg dose of SB-705498 had any effect on the temperature threshold for GTCS induced by hyperthermia (Figure 2A). In order to confirm sufficient exposure to SB-705498 for Trpv1 inhibition, the brain SB-705498 concentrations in experimental animals were measured. The brain concentrations of SB-705498 following 10 and 20 mg/kg treatments were 2.1 ± 0.5 ng/mg brain (4.9 µM) and 2.4 ± 1.1 ng/mg brain (5.6 µM), respectively with *n* = 6 per group. These concentrations are ∼800–900 times higher than the IC_50_ of SB-705498 at Trpv1 channels against a heat stimulus (6 nM) (Gunthorpe et al., 2007). Even assuming high protein binding of SB-705498 resulting in a free-fraction of 1%, the brain concentrations of SB-705498 would still be 8–9 times higher than the IC_50_. Thus, lack of seizure protection was not due to subtherapeutic brain exposure.

Next, we examined the effects of Trpv1 receptor inhibition on spontaneous seizures of F1.*Scn1a* ^*+/−*^ mice. SB-705498 was administered subchronically *via* supplementation in chow (500 mg SB-705498/kg chow). SB-705498 treatment did not affect spontaneous seizure frequency (Figure 2B). While SB-705498 treatment trended toward increasing the proportion of mice experiencing GTCS compared to control treatment (56.3% vs. 88.9%, respectively), this was not statistically significant (*p* = 0.052) (Figure 2B). The severity of spontaneous seizures as measured by the proportion of GTCS with full hindlimb extension was also comparable in control chow (Figure 2C) and 500 mg SB-705498/kg chow treated mice. SB-705498 did not affect survival of F1.*Scn1a* ^*+/−*^ mice (Figure 2D). The brain levels of SB-705498 with oral supplementation was 0.68 ± 0.19 ng/mg brain (263 times higher than the IC_50_, Supplementary Table S1), this rules out the lack of efficacy due to sub-optimal dosing.

### Heterozygous deletion of *Trpv1* in F1.*Scn1a* ^*+/−*^ mice increased sensitivity to hyperthermia-induced seizures at P18 but reduced spontaneous seizure severity

**FIGURE 3:**
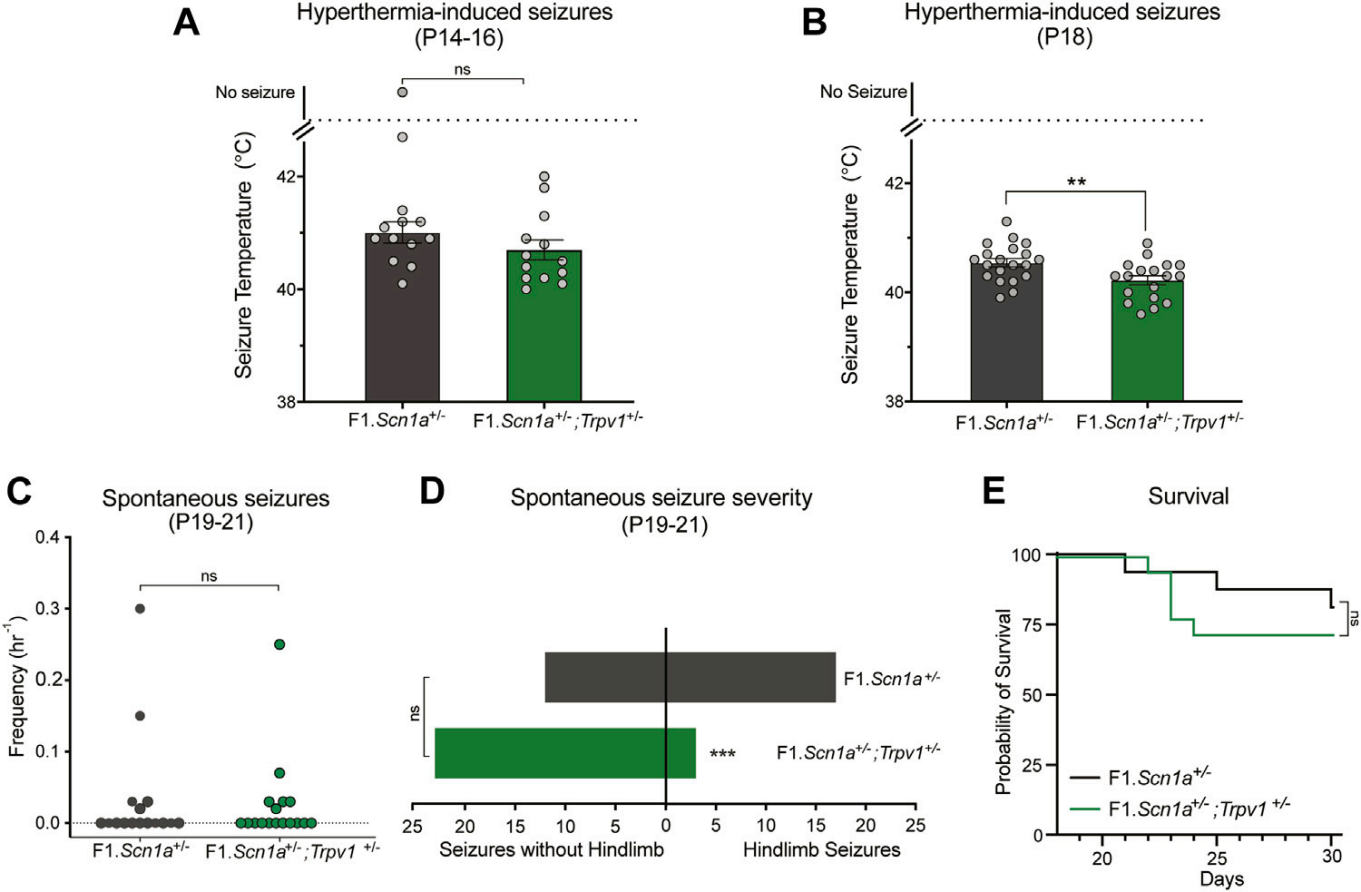
*Trpv1* deletion had both pro and anti-convulsant effects in the F1.*Scn1a* ^*+/−*^ mice. **(A)** The GTCS temperature threshold of P14-16 in F1.*Scn1a* ^*+/−*^ mice (41.20 ± 0.9°C) and were similar to that of F1.*Scn1a* ^*+/−*^; *Trpv1* ^+/−^ mice (40.70 ± 0.6°C; *n* = 13 per group; *p* = 0.15; unpaired *t*-test). Bars represent the average temperatures of seizure induction and error bars represent SEM. **(B)** Effects of heterozygous loss of *Trpv1* on spontaneous seizures was determined in a separate cohort of mice. Loss of *Trpv1* had a pro-convulsant effect at P18 as it reduced the GTCS temperature threshold in F1.*Scn1a* ^*+/−*^; *Trpv1* ^+/−^ mice (40.5 ± 0.07, *n* = 20) compared to F1.*Scn1a* ^*+/−*^ mice (40.2 ± 0.08, *n* = 18; *p* = 0.007; unpaired *t*-test). **(C)** Frequency of spontaneous GTCS of individual F1.*Scn1a* ^*+/−*^ mice and F1.*Scn1a* ^*+/−*^; *Trpv1* ^+/−^ mice. Loss of *Trpv1* did not alter the spontaneous seizure frequency in P19–21 F1.*Scn1a* ^*+/−*^; *Trpv1* ^+/−^ mice (0.023 ± 0.01/h, *n* = 18) compared to F1.*Scn1a* ^*+/−*^ mice (0.033 ± 0.02/h, *n* = 16; *p* = 0.7; unpaired *t*-test). **(D)** Severity of spontaneous seizures, i.e., the number of GTCS with and without full tonic hindlimb extension is depicted. Seizure severity was reduced in F1.*Scn1a* ^*+/−*^; *Trpv1* ^+/−^ mice, 11.5% of GCTS (3 of 26) resulted in full hindlimb extension, while in F1.*Scn1a* ^*+/−*^ mice 58.6% (17 of 29) of GTCS had full hindlimb extension (*p* < 0.001, Fisher’s exact test). **(E)** Survival curves comparing survival until P30. Survival was not altered in F1.*Scn1a* ^*+/−*^; *Trpv1* ^+/−^ mice (72.2%) relative to that of F1.*Scn1a* ^*+/−*^ mice [81.3%; *p* = 0.52, Log-rank (Mantel-Cox) test].

Pharmacological validation studies may suffer from selectivity issues due to unanticipated off-target effects. Arguably, a genetic approach is a more selective method for target validation. Accordingly, we crossed *Trpv1* ^*+/−*^ mice with *Scn1a* ^*+/−*^ mice, to assess whether partial genetic deletion of *Trpv1* affected DS phenotypes of *Scn1a* ^*+/−*^ mice. Heterozygous deletion of *Trpv1* had no effect on hyperthermia-induced seizures of F1.*Scn1a* ^*+/−*^ mice at P14–16. The temperature threshold for hyperthermia-induced GTCS was not different between F1.*Scn1a* ^*+/−*^;*Trpv1* ^*+/−*^ mice and F1.*Scn1a* ^*+/−*^ mice (Figure 3A). Surprisingly, when mice were subjected to a single and brief hyperthermia-induced GTCS at P18 prior to monitoring spontaneous seizures, an effect of *Trpv1* expression was observed. F1.*Scn1a* ^*+/−*^;*Trpv1* ^*+/−*^ had a significantly lower temperature threshold compared to F1.*Scn1a* ^*+/−*^ mice (Figure 3B) indicating a proconvulsant effect. However, the frequency of spontaneous seizures and proportion of mice exhibiting GTCS was not different between F1.*Scn1a* ^*+/−*^;*Trpv1* ^*+/−*^ mice and F1.*Scn1a* ^*+/−*^ mice (Figure 3C). Interestingly, the severity of spontaneous seizures (i.e., the proportion of GTCS with full hindlimb extension) was significantly reduced in F1.*Scn1a* ^*+/−*^; *Trpv1* ^*+/−*^ mice (Figure 3D). F1.*Scn1a* ^*+/−*^; *Trpv1* ^*+/−*^ mice had three hindlimb seizures out of 26 (11.5%); whereas, F1.*Scn1a* ^*+/−*^ mice had 17 hindlimb seizures out of 29 (58.6%). Although the severity of spontaneous seizures was reduced with *Trpv1* heterozygous deletion, survival of F1.*Scn1a* ^*+/−*^;*Trpv1* ^*+/−*^ mice was not different from F1.*Scn1a* ^*+/−*^ mice (Figure 3E). However, survival of the F1.*Scn1a* ^*+/−*^ mice in this cohort was high (81%) compared to the typical ∼50% survival of *Scn1a* ^*+/−*^ mice (Mistry et al., 2014; Hawkins et al., 2017; Han et al., 2020), which may have created a ceiling effect. Although the *Trpv1* ^*+/−*^ mice are congenic on C57BL/6J, 129ES cells were used in the generation of these mice, and this line still carries residual 129 alleles in their genome (Supplementary Figure S3). Some of the residual 129 overlaps a modifier locus shown to influence survival in F1.*Scn1a* ^*+/−*^ mice (Miller et al., 2014; Hawkins and Kearney, 2016; Calhoun et al., 2017). Furthermore, survival of F1.*Scn1a* ^*+/−*^ mice is known to vary across laboratories and even across cohorts within the same laboratory (Anderson et al., 2019, 2020; Benson et al., 2020).

## Discussion

Emerging evidence suggests Trpv1 mediates the anticonvulsant effects of the phytocannabinoid CBD (Vilela et al., 2014; Gray et al., 2020). Moreover, Trpv1 has been characterized as a novel anticonvulsant drug target in conventional animal seizure models (Zavala-Tecuapetla et al., 2020). However, whether this channel is a viable target in animal models of drug-resistant epilepsy is unknown. Using pharmacological and genetic approaches we examined whether Trpv1 contributes to the seizure phenotype of the *Scn1a* ^*+/−*^ mouse model of DS, a severe intractable childhood epilepsy. We found that *Trpv1* mRNA expression was higher in seizure-susceptible F1 mice compared to seizure resistant 129 mice, which suggests Trpv1 is a candidate genetic modifier in DS mice. The selective Trpv1 antagonist SB-705498 did not significantly affect hyperthermia-induced seizures, spontaneous seizures, or survival. However, heterozygous deletion of *Trpv1* in combination with *Scn1a* had mixed effects. Heterozygous *Trpv1* deletion was proconvulsant against hyperthermia-induced seizures in DS mice but had anticonvulsant effects on spontaneous seizures by reducing seizure severity.

Pharmacological inhibition of the Trpv1 receptor or genetic deletion of *Trpv1* has anticonvulsant effects in both chemically and electrically induced seizures in adult rodents (Chen et al., 2013; Gonzalez-Reyes et al., 2013; Jia et al., 2015; Socała et al., 2015). Our results here in the DS mouse model of intractable epilepsy reveals a more complicated picture, with pharmacological inhibition of Trpv1 failing to yield anticonvulsant effects, and heterozygous deletion of *Trpv1* having only modest and conflicting anticonvulsant and proconvulsant effects dependent on the type of seizure examined. Collectively, these results are consistent with *Trpv1* being a genetic modifier in the *Scn1a* ^*+/−*^ mice, albeit with a subtle modifying effect on the seizure phenotype.

Heterozygous deletion of *Trpv1* in F1.*Scn1a* ^*+/−*^ mice had no effect on the seizure temperature threshold from P14-16 but had pro-convulsant effects against hyperthermia-induced seizures at P18, an age at which the spontaneous seizures appear in F1.*Scn1a* ^*+/−*^ mice (Figure 3B). One plausible reason for this is based on the developmental trajectory of Trpv1 expression in the brain. In C57BL/6J mice, Trpv1 mRNA and protein expression correlates well and is relatively low at P14-16; however, mRNA and protein levels significantly increase by P28 and peak at P56 in the cortex and hippocampus (Huang et al., 2014). This implies that during the P14-16 testing window Trpv1 expression is likely too low to affect hyperthermia-induced seizures. While from P18, Trpv1 expression has likely reached a sufficient level to enable the effects of *Trpv1* deletion to be manifest. Future studies might interrogate the role of Trpv1 in DS mouse phenotypes in older adolescent and adult mice to observe whether the effects of Trpv1 can be magnified. The proconvulsant effects of Trpv1 deletion on febrile seizures may not be *Scn1a*-specific, as *Trpv1* deletion exacerbated hyperthermia-induced seizures in wild-type C57BL/6J mice (Barrett et al., 2016). Consistent with our results, this effect was age-dependent, with the effect being observed at P20 but not at P15. This prior study also observed that *Trpv1* ^*−/−*^ mice displayed a rapid rise in body temperature hinting that impaired thermoregulation may be involved in triggering the seizures.

Our genetic validation experiments suggest that Trpv1 has opposing roles on thermally induced vs. spontaneous seizures. These opposing effects are not surprising considering the accumulating evidence of various drugs having dissociable effects on these different types of seizures. For example, treatment of F1.*Scn1a* ^*+/−*^ mice with a combination of the phytocannabinoids Δ^9^-tetrahydrocannabinol (THC) and CBD yielded anticonvulsant effects against hyperthermia-induced seizures but had proconvulsant effects on spontaneous seizure severity (Anderson et al., 2020). Moreover, stiripentol was anticonvulsant against hyperthermia-induced seizures but not spontaneous seizures; whereas, the opposite effects were seen with the novel sodium channel blocker GS967, which did not affect hyperthermia-induced seizures but reduced spontaneous seizures (Anderson et al., 2017; Hawkins et al., 2017).

Consistent with anticonvulsant effects of *Trpv1* deletion in conventional models of induced seizures (Barrett et al., 2016; Gray et al., 2020), our results provide the first evidence that heterozygous deletion of *Trpv1* can reduce seizure severity in a genetic mouse model of childhood epilepsy. Our results suggest that Trpv1 receptors may not participate in the generation of spontaneous seizures but rather affect the severity of seizures once they are initiated. This result accords with evidence showing that Trpv1 receptor activation enhances neuronal excitability *via* cation influx that triggers the release of the excitatory neurotransmitter glutamate (Bhaskaran and Smith, 2010; Peters et al., 2010; Shoudai et al., 2010).

It is difficult to explain why pharmacological inhibition of Trpv1 with SB-705498 did not mirror the findings of partial genetic deletion of *Trpv1* on seizure phenotypes in F1.*Scn1a* ^*+/−*^. Our results suggest genetic deletion of *Trpv1* had a greater impact on disrupting Trpv1 function than pharmacological blockade, or that the germline deletion of *Trpv1* evokes compensatory molecular changes that contributes to the observed results. The brain concentrations of SB-705498 following i.p. injection or oral delivery were in excess of the concentrations necessary to inhibit heat-induced activation of Trpv1 *in vitro* (Gunthorpe et al., 2007). Moreover, the i.p. doses of 10 and 20 mg/kg SB-705498 encompass doses reported to be effective *in vivo* in mice (Van Den Wijngaard et al., 2009; Tékus et al., 2010). Thus, the lack of effect is unlikely explained by SB-705498 not attaining sufficiently high brain concentrations to engage Trpv1 receptors.

We chose SB-705498 because it is a selective Trpv1 antagonist (Rami et al., 2006) which doesn’t bind a diverse array of receptors, channels and enzymes, as well as having >100-fold selectivity at TRPV1 over TRPM8 (Gunthorpe et al., 2007). Moreover, SB-705498 has favorable pharmacokinetic properties with good oral bioavailability, which in part supported its clinical development (Chizh et al., 2007; Kym et al., 2009). It is noteworthy that most pharmacological validation studies of Trpv1 in seizure models used capsazepine, a drug known to have off-target effects leading to misleading findings (Ducrocq et al., 2019). Unlike SB-705498, capsazepine has equivalent inhibitory effects on TRPV1 and TRPM8 (Weil et al., 2005; Gunthorpe et al., 2007). Interestingly, the selective Trpv1 antagonist SB-366791, which does not have appreciable effects at TRPM8 like SB-705498, did not significantly influence PTZ-induced seizures (Weil et al., 2005; Vilela et al., 2014).

In conclusion, our results suggest that *Trpv1* expression modifies the spontaneous seizure severity of F1.*Scn1a* ^*+/−*^ mice. Heterozygous *Trpv1* deletion had modest and conflicting effects on the seizure phenotype of the F1.*Scn1a* ^*+/−*^ mice; its deletion exerted anticonvulsant effects by reducing spontaneous seizure severity, although it was proconvulsant against hyperthermia-induced seizures. This latter effect highlights a major potential liability for the development of novel Trpv1 inhibitors. Further reinforcing the view that Trpv1 inhibitors may be a therapeutic dead-end for treating DS, the selective inhibitor of Trpv1 receptors, SB-705498, failed to ameliorate seizure phenotypes in the F1.*Scn1a* ^*+/−*^ mice. Collectively, these data favor the conclusion that Trpv1 is not a viable drug target for developing new anticonvulsant drugs for DS, an intractable childhood epilepsy.

## Data Availability

The raw data supporting the conclusions of this article will be made available by the authors, without undue reservation.
