# Supplementary material for: The Heat Sensing Trpv1 Receptor Is Not a Viable Anticonvulsant Drug Target in the Scn1a +/− Mouse Model of Dravet Syndrome
Source: Front Pharmacol. 2021 May 17;12:675128. doi: 10.3389/fphar.2021.675128 (PMC8165383; doi:10.3389/fphar.2021.675128)
Supplement: Supplementary file 1 [file Table1.docx]

| SB-705498 dose | Brain levels (ng/mg) | Brain levels (µM) |
| --- | --- | --- |
| 10 mg/kg i.p. | 2.06 ± 0.53 | 4.79 ± 1.24 |
| 20 mg/kg i.p. | 2.40 ± 1.14 | 5.58 ± 2.65 |
| 500 mg/kg chow | 0.68 ± 0.19 | 1.58 ± 0.45 |
|  |  |  |

**Supplemental Table 1: Brian levels of SB-705498 after seizure testing.** Brain concentration of SB-705498 in F1.*Scn1a^+/-^* mice were measured after hyperthermia-induced seizure testing with i.p. doses of 10mg/kg (n=6) or 20 mg/kg (n=6) and after spontaneous seizure testing with 500mg/kg chow of oral dose (n=6). The brain samples were randomly chosen from the animals presented in Fig 2. Both dosing strategies resulted in brain levels several fold higher than the IC_50_ of Trpv1 receptors for the heat stimulus (6 nM or 2.58 pg/mg). LC-MS/MS was used to measure brain levels as described in the method section.
