# Supplementary figures and images for: The Heat Sensing Trpv1 Receptor Is Not a Viable Anticonvulsant Drug Target in the Scn1a +/− Mouse Model of Dravet Syndrome
Source: Front Pharmacol. 2021 May 17;12:675128. doi: 10.3389/fphar.2021.675128 (PMC8165383; doi:10.3389/fphar.2021.675128)

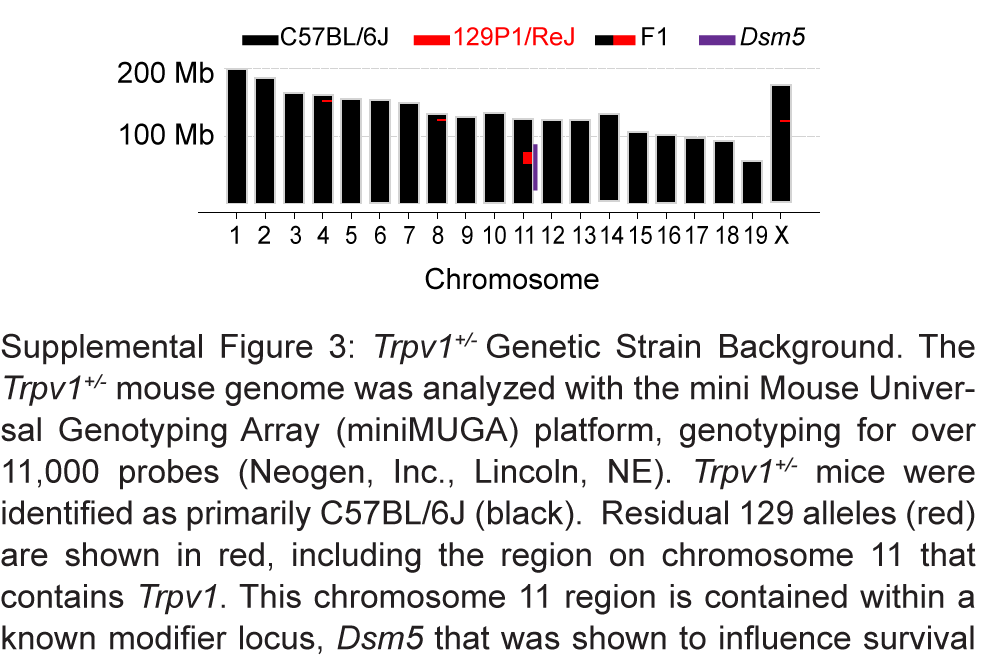

Supplement: Supplementary file 2 [file Image3.tif]

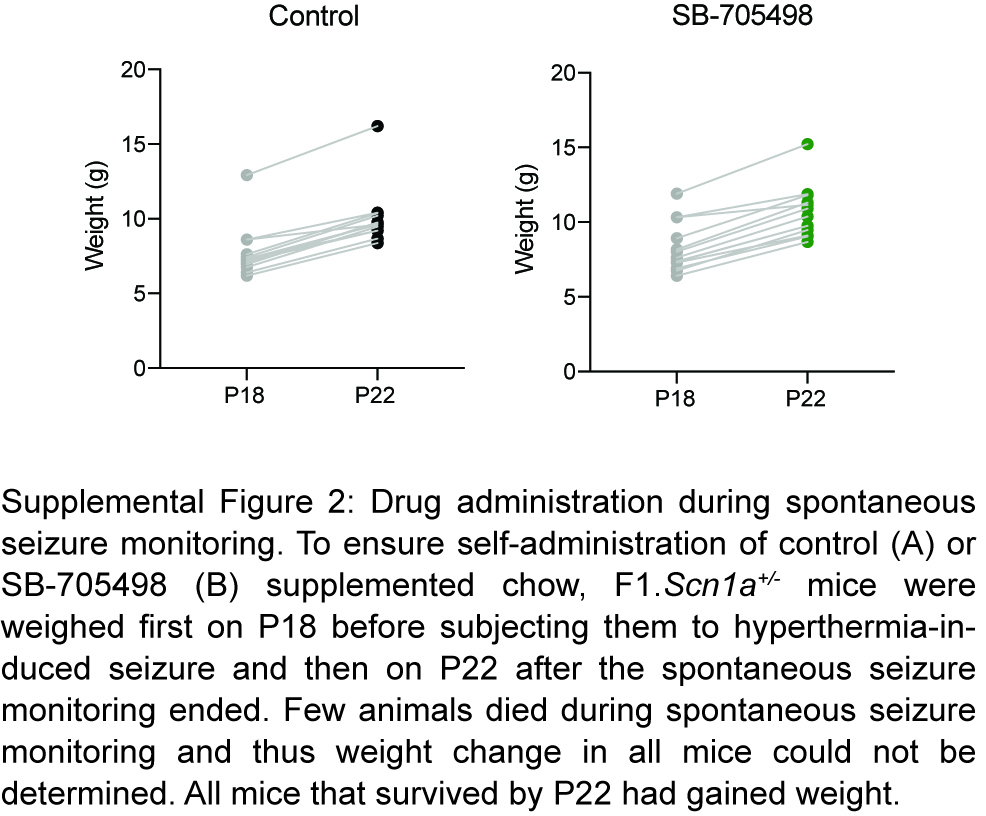

Supplement: Supplementary file 3 [file Image2.tif]

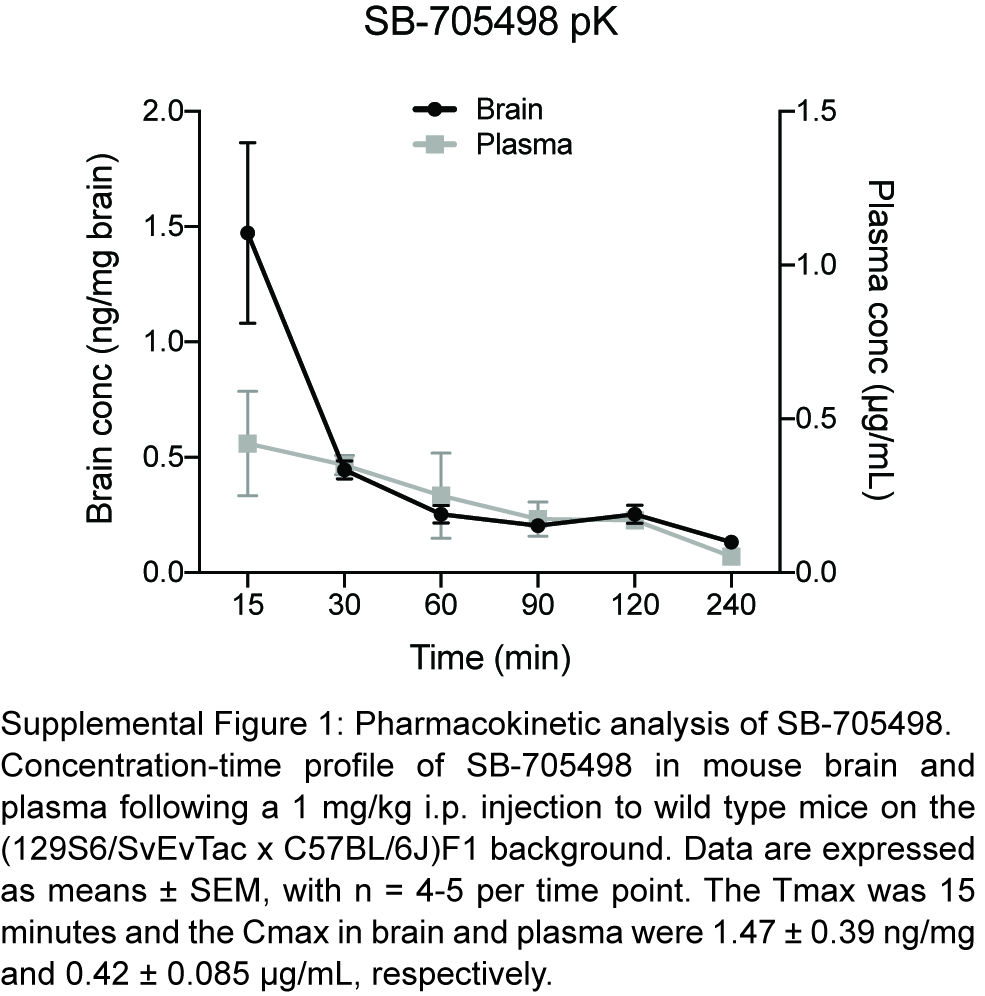

Supplement: Supplementary file 4 [file Image1.tif]
